# Proteomic Analysis of Secretomes of Oncolytic Herpes Simplex Virus-Infected Squamous Cell Carcinoma Cells

**DOI:** 10.3390/cancers10020028

**Published:** 2018-01-23

**Authors:** Shinya Tada, Masakazu Hamada, Yoshiaki Yura

**Affiliations:** Department of Oral and Maxillofacial Surgery, Osaka University Graduate School of Dentistry, Suita, Osaka 565-0871, Japan; dt3034ts@yahoo.co.jp (S.T.); hmdmskz@dent.osaka-u.ac.jp (M.H.)

**Keywords:** oncolytic virotherapy, herpes simplex virus, secretome, proteomics

## Abstract

Oncolytic herpes simplex virus type 1 (HSV-1) strain RH2 induced immunogenic cell death (ICD) with the release and surface exposure of damage-associated molecular patterns (DAMPs) in squamous cell carcinoma (SCC) SCCVII cells. The supernatants of RH2-infected SCCVII cells also exhibited antitumor ability by intratumoral administration in SCCVII tumor-bearing mice. The supernatants of RH2-infected cells and mock-infected cells were concentrated to produce Med24 and MedC for proteomic analyses. In Med24, the up- and down-regulated proteins were observed. Proteins including filamin, tubulin, t-complex protein 1 (TCP-1), and heat shock proteins (HSPs), were up-regulated, while extracellular matrix (ECM) proteins were markedly down-regulated. Viral proteins were detected in Med 24. These results indicate that HSV-1 RH2 infection increases the release of danger signal proteins and viral gene products, but decreases the release of ECM components. These changes may alter the tumor microenvironment (TME) and contribute to enhancement of anti-tumor immunity against SCC.

## 1. Introduction

Oncolytic virotherapy is a novel therapeutic modality that directly induces the lysis of infected tumor cells, and subsequently enhances host immune responses [1,2,3,4]. Recent clinical studies demonstrated that oncolytic viral therapy increases cytokines, activates immune responses, and effectively destroys primary and metastatic lesions, even if the virus does not reach distant lesions. Therefore, oncolytic viruses are also considered to be immunotherapeutic agents [5]. Herpes simplex virus type 1 (HSV-1) is one of the most widely studied viruses for the treatment of patients with solid tumors. Oncolytic HSV-1 lacks the neurovirulence gene γ34.5, which is responsible for encephalitis, as a requisite gene modification for safety [6,7].

In microbial infection, pathogen-associated molecular patterns (PAMPs) that exist in diverse organisms, but not in the host, provide exogenous signal regarding the presence of pathogens in the immune system, thereby promoting immunity [8,9]. In contrast, cells release damage-associated molecular patterns (DAMPs) as endogenous signals that alert the immune system to respond to unexpected cell death, microbial invasion, and stress [3,10]. DAMPs may be proteins, nucleic acids, or metabolic products. Protein DAMPs include intracellular proteins such as high mobility group box 1 (HMGB1), heat shock protein (HSP), hyaluronic acid, calreticulin (CRT), and S100 protein [3,5]. When solid tumors are infected with oncolytic viruses, the virus mostly induces immunogenic cell death (ICD), resulting in the cell surface exposure of CRT and HSP and release of ATP, HMGB1, uric acid, other DAMPs, PAMPs, and tumor-associated antigens (TAAs) [3,11,12,13].

Previously, we reported that an injection of oncolytic HSV-1 strain RH2 into squamous cell carcinoma cell (SCC) SCCVII tumors in inbred mice enhanced systemic anti-tumor immunity [14], and that cell death caused by RH2 was immunogenic cell death (ICD) with the release of DAMPs such as ATP and HMGB1 [13]. We also indicated that intratumoral injection of the supernatants of RH2-infected cells suppressed tumor growth, even if infectivity was lost by ultraviolet irradiation, suggesting their ability to enhance antitumor immunity [13].

Secreted proteins comprise an important group of molecules encoded by about 10% of the human genome, and are able to reflect a broad variety of different conditions of the cell [15]. A proteomic analysis of human macrophages infected with HSV-1 demonstrated the release of proteins functionally related to metabolic processes, transport, stress responses, cell death, proteolysis, the extracellular matrix (ECM), and cell adhesion [16]. However, the characteristics of the secreted proteins, secretomes, of oncolytic HSV-1-infected tumor cells have not yet been studied. In the present study, we performed proteomic analysis of ecretomes of RH2-infected and mock-infected SCC cells, and compared the protein profiles.

## 2. Results

### 2.1. Classification of Released Proteins of Med24 and MedC

The supernatants of RH2-infected SCCVII cells and mock-infected cells were harvested and concentrated to produce samples named Med24 and MedC. Med24 and MedC were sequentially analyzed, and isobaric tag for relative and absolute quantitation (iTRAQ) quantitation was performed for each protein. A total of 1567 proteins were detected in Med24 and 1344 in MedC, with 223 extracellular proteins being increased by RH2 infection. Based on the results of iTRAQ quantitation, MedC proteins spontaneously secreted from SCCVII cells were classified into three categories, i.e., high, medium, and low abundance groups. The numbers of proteins in the high, medium, and low abundance groups were 190, 262, and 892, respectively.

In order to examine the effects of RH2 infection on protein secretion, iTRAQ quantitation data for each protein was compared between Med24 and MedC. Protein release was found to be up- or down-regulated by RH2 infection. In the high abundance group, 29 proteins were up-regulated by more than 1.4 fold by RH2 infection (Table 1). However, a significant increase was observed in 16 proteins. They included filamin alpha, tubulin beta-4B, clathrin heavy chain, t-complex protein 1 (TCP-1) subunit beta, theta, alpha, gamma and delta, bifunctional purine biosynthesis protein (PURH), filamin-C, bifunctional glutamate/proline–tRNA ligase, plastin-3, fascin, coatomer subunit alpha, beta enolase, and tubulin beta-6 chain.

In the high abundance group of MedC, 50 proteins were down-regulated by more than 50% by RH2 infection (Table 2). They included ECM components such as fibronectin, thrombospodin-1, basement membrane-specific heparin sulfate proteoglycan core protein, fibrillin-1, chondroitin sulfate proteoglycan 4, SPARC, procollagen C, collagen alpha, and extracellular matrix protein 1. Other proteins, cathepsin B, cathepsin D, matrixmetalloproteinase-9, and 72 kDa type IV collagenase, were involved in the remodeling of ECM. Among all proteins listed, a significant difference was observed between the Med24 and MedC groups.

### 2.2. Alteration of DAMPs and DAMPs-Related Proteins in the Secretomes

In DAMPs, the numbers of high, medium, and low abundance proteins were 11, 6, and 3, respectively (Table 3). In the high abundance group, HSPs, actin cytoplasmic 1, annexin A1, annexin A2, galectin-1, peroxiredoxin-2, and histone H2B type 1 were observed. Only the levels of HSP90-beta, actin cytoplasmic 1, and galectin-3 were significantly higher in Med24 than in MedC. ATP-citrate, which is responsible for the production of ATP, a known DAMP, was also detected as a significantly elevated protein (Table 3).

### 2.3. Viral Proteins in the Secretomes

Twenty-nine viral proteins that were detected in Med24 were not present in MedC (Table 4). When viral proteins were categorized into three groups, the numbers of proteins in the high, medium, and low abundance groups were 8, 9, and 12, respectively. Major capsid protein unique sequence of the L component (UL) 19, tegument protein UL37, capsid protein virion polypeptide (VP) 23, glycoprotein D, and glycoprotein E were identified as viral structural proteins. In addition, ribonucleoside-diphosphate reductase, single-strand DNA-binding protein, DNA polymerase processivity subunit, transcriptional regulator infected cell protein (ICP) 4, exonuclease (UL12), multifunctional expression regulator (ICP27), and deoxyuridine triphosphatase were identified as high abundance proteins in Med24 (Table 4).

## 3. Discussion

Proteomic analysis has been applied to HSV-1 virion-incorporated host proteins, cellular proteins interacting with viral proteins such as ICP27, ICP8, and VP16, and protein profiles in HSV-1-infected cells [17,18,19]. Miettinen et al. [16] characterized the secretome of HSV-1-infected human primary macrophage using high-throughput quantitative proteomics, and identified 516 distinct proteins from macrophages secretome upon HSV-1 infection, and the secretion of 411 proteins was >2-fold increased upon interferon β (IFN-β) priming and/or HSV-1 infection. Based on our previous findings that the supernatants of RH2-infected cells suppressed the tumor growth in mice [13], we hypothesized that the supernatants contained immunostimulatory proteins, and performed proteomic analysis of the secretomes. Since the amounts of proteins detectable in the secretomes were important, we classified MedC proteins into three categories based on the results of iTRAQ quantitation, and then determined whether they were up-regulated or down-regulated by RH2 infection. When the high abundance proteins that increased by more than 1.4 fold following RH2 infection were selected, 29 proteins fulfilled this criterion. However, a significant increase was only observed in 16 proteins, including filamin alpha, tubulin beta-4B, clathrin heavy chain, and TCP-1s (Table 1).

Actin-binding proteins, filamin and pastin-3, and the actin-bundling protein, fascin, were identified as up-regulated high abundance proteins. An increase in tubulin, which is involved in the polymerization of microtubules, was demonstrated. Autoantibodies against filamin C were elevated in the patients with low-grade glioma and those against tubulin increased in the patients with nasopharyngeal carcinoma and neuroblastoma [20,21,22]. Clathrin heavy chain is one of the most important proteins known to be involved in the secretion and transport of vesicles [23]. Seliger et al. [24] examined potential candidate biomarkers and novel targets for T-cell-based immunotherapies of renal carcinoma, and identified human leukocyte antigen class I ligands, including clathrin heavy chain. Filamin, tubulin, and clathrin may be potential immunogenic antigens in specific cancers.

We also identified TCP-1 family members as the major up-regulated proteins in Med24 secretomes. Chaperonin-containing TCP-1 (CCT), known as the TCP-1 ring complex (TRiC), forms an oligomer that consists of distinct subunits (alpha, beta, gamma, delta, epsilon, zeta, eta, and theta) occupying a fixed position within the two back-to-back chaperonin rings [25]. CCT was originally identified as a molecular chaperone that is required for the folding of the highly abundant cytoskeletal proteins, actin and tubulin [26]. The genes for CCT alpha and CCT beta were amplified in breast cancer and necessary for cancer growth and proliferation [27]. CCT beta and CCT epsilon were overexpressed in both hepatocellular and colorectal cancers [28,29]. Gao et al. [30] reported that CCT5 (CCT epsilon) induced an autoantibody response in non-small cell lung cancer sera, and showed higher expression in cancer tissues. In the case of melanoma immunotherapy, mutated TCP 1-zeta-6A (CCT6A) antigen had previously been identified and proved to be immunogenic in a melanoma patient, so mutated CCT6A peptide was used as a model for patient-specific neoantigen [31]. Therefore, TCP-1 subunits identified in the present study may act as TAAs required for capable of activating tumor-specific immunity.

In secretomes of HSV-1-infected macrophages, endogenous danger signal proteins were demonstrated as up-regulated proteins. They included HSPs, annexins, S100-A proteins, galectins, and thioredoxin superfamily members [16]. The HSP family comprises proteins that act as molecular chaperones and catalyze the folding of proteins. HSPs bind to potential antigens under necrotic conditions and deliver them to various antigen-presenting cells [32]. We found 20 proteins that are known as DAMPs (Table 3). High abundance DAMPs including heat shock cognate 71 kDa protein, HSP90-alpha, HSP 70 kDa protein 4, HSP60, annexin A1, and peroxiredoxin-2 increased by RH2 infection, but the fold change observed was less than 1.4 fold. The levels of HSP90-beta, actin cytoplasmic 1, and galectin-3 were significantly higher in Med24 than in MedC. ATP is a known DAMP released extensively during ICD [4]. We also confirmed that ATP-citrate synthase and ATP synthase were up-regulated proteins as DAMPs-related proteins. The spatiotemporally coordinated emission of specific DAMPs promotes the recruitment of antigen presenting cells to sites of ongoing ICD, their ability to take up dead cell-derived particulate materials, as well as their capacity to prime an adaptive immune response [33]. Up-regulated DAMPs and DAMPs-related proteins observed in the present study were considered to be involved in the augmentation of antitumor immunity, operating as adjuvants.

Exosomes are small membrane-derived vesicles secreted by many types of normal and tumor cells [34]. Xiang et al. [35] reported the presence of DAMPs, such as HSPs, annexins, and histones, in exosomes, indicating the role of microvesicles as a form of excretion. DAMPs observed in the secretomes of MedC may be partly contained in exosomes. However, since oncolytic viruses including RH2 have been shown to induce apoptosis, necroptosis, autophagy, and pyroptosis [3,5,36,37,38], increases in the secretion of DAMPs may be mostly due to the disintegration of the cell membrane by virus-mediated cell death.

Another result of the present study is the marked decrease in the release of proteins constituting the tumor microenvironment (TME). In the present study, 50 proteins were selected as down-regulated proteins that were reduced to less than 0.5 (Table 2). They included fibronectin, thrombospondin-1, basement membrane-specific heparan sulfate proteoglycan core protein, fibrillin-1, 72 kDa type IV collagenase, procollagen C, and extracellular matrix protein 1. Since tumor and tumor stroma create the TME, which efficiently promotes tumor progression and supports evasion from antitumor immunity [39,40], oncolytic HSV-1 may exert its antitumor effects by decreasing the release of ECM components.

Using the secretomes of HSV-1-infected macrophages, 5 envelope proteins, 3 tegument proteins, alkaline nuclease, and deoxyuridine 5′-triphosphate nucleotidohydrolase were identified [16]. In the present study, viral structural components, i.e., the tegument, nucleocapsid, and envelope, were also detected in the supernatants of HSV-1-infected SCCVII cells. Furthermore, viral enzymes responsible for viral replication such as ribonucleoside-diphosphate reductase, single-strand DNA-binding protein, DNA polymerase processivity subunit, deoxyuridine triphosphatase, and thymidine kinase were identified. This result indicated that these cytoplasmic and nuclear viral proteins were passively released from dying cells. These proteins have been suggested to act as PAMPs, which promote inflammatory reactions and induce the infiltration of innate and adaptive immune cells.

**Figure 1:**
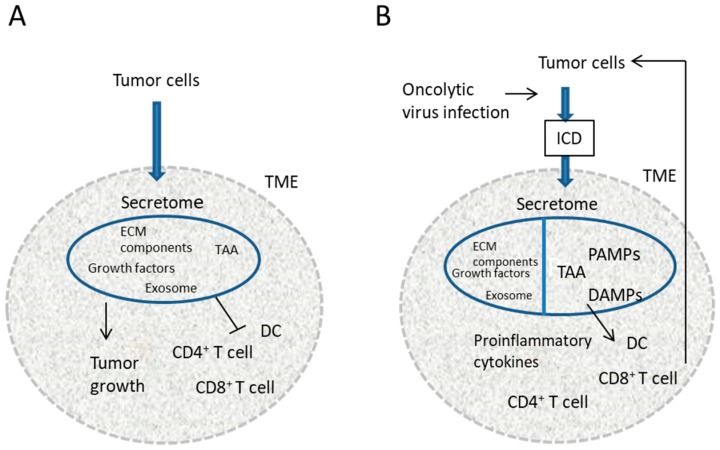
A model of the augmentation of antitumor immunity by an oncolytic virus. (**A**) Immunological abnormalities associated with TME inhibit the priming of antitumor adaptive immunity or tolerize tumor-specific CD4^+^ and CD8^+^ T cells; (**B**) Oncolytic viruses induce ICD with the release of secretomes containing DAMPs, PAMPs, proinflammatory cytokines, and TAA. This triggers DC maturation and cytotoxic CD8^+^ T cell proliferation. The reduced secretion of stromal components due to viral infection also overcomes TME-associated immunosuppression. TME, tumor microenvironment; ECM, extracellular matrix; TAA, tumor-associated antigen; ICD, immunogenic cell death; DC, dendritic cell; PAMPs, pathogen-associated molecular patterns; DAMPs, damage-associated molecular patterns.

Immunological abnormalities associated with TME inhibit the priming of antitumor adaptive immunity or tolerize tumor-specific CD4^+^ and CD8^+^ T cells [40,41] (Figure 1A). Oncolytic virotherapy is supposed to release DAMPs, PAMPs, and TAA triggering proinflammatory cytokine release, DC maturation, and cytotoxic CD8^+^ T cell proliferation, and overcome TME-associated immunosuppression by reducing secretion of stromal components [5,40,42,43] (Figure 1B). Using the supernatants of RH2-infected SCC cells, we herein demonstrated that DAMPs increased, and ECM proteins decreased. Viral gene products were also included. This may promote the immunological effects of the oncolytic HSV-1 on SCC cells. As a next step, it is important to confirm the role of each secreted protein in the antitumor effects of oncolytic HSV-1 by specific deletion.

## 4. Materials and Methods

### 4.1. Cells and Virus

SCCVII cells derived from the cutaneous SCC of C3H mice were cultured with Eagle’s minimal essential medium supplemented with 10% calf serum at 37 °C in a humidified atmosphere with 5% CO_2_ [13,14]. HSV-1 RH2 lacking the γ34.5 gene and with multiple mutations including glycoprotein B were grown in Vero cell monolayers and infectivity was assessed using a plaque assay on a Vero cell monolayer [14,44].

### 4.2. Preparation of Concentrated Supernatants of RH2-Infected Cells

SCCVII cells were infected with RH2 at a multiplicity of infection of 10. After being incubated for 60 min for virus adsorption, cell monolayers were washed twice with phosphate-buffered saline, covered with serum-free medium, and cultured for 24 h. Culture supernatants were then harvested, and centrifuged at 1500 rpm for 10 min to remove cell debris. The supernatants were concentrated 30 times using Amicon^®^Ultra-15 3 K Centrifugal Filter Devices (Merck, Darmstadt, Germany) [13]. Concentrates were exposed to ultraviolet irradiation at an intensity of 0.15 mW/cm^2^ for 30 min in order to inactivate the infectivity of the virus, filtered through a 0.20-µm filter, and named Med24. Mock-infected cells were also incubated in serum-free medium, supernatants were harvested 24 h later, treated as described for RH2-infected cells, and named MedC.

### 4.3. Proteomic Analysis

Three samples were used each in the proteomic analysis of Med24 and MedC [45]. Protein concentrations in Med24 and MedC were assessed, and 4 volumes of cold acetone per 100 µg were added to each sample. They were kept at −20 °C overnight, and centrifuged at 15,000 rpm for 10 min to collect precipitates. Proteins were dried, and denatured by resuspending in 20 µL of lysis buffer (50 mM Tris-HCl, pH 9.0, 6 M uric acid, and 5% sodium deoxycholate), and reduced with 2 µL of reduction buffer (10 mM dithiothreitol) at 37 °C for 1 h. In order to block cysteine, 1 µL of cysteine-blocking buffer (55 mM iodoacetamide) was added, and proteins were alkylated in the dark at 25 °C for 30 min. After a 10-fold dilution of samples with 50 mM Tris-HCl (pH 9.0), proteins were disintegrated with trypsin at 37 °C for 16 h. An equal volume of ethyl acetate was added and acidified to a final concentration of 0.5% trifluoroacetic acid. The aqueous solution after centrifugation at 14,000 rpm for 2 min was harvested. Samples were desalted on C18-Stage Tips and labeled with the iTRAQ tag dissolved in 70 µL ethanol at room temperature for 1 h.

In the LC-MS/MS analysis, the Ultimate 3000 Nano LC system (Thermo Fisher Scientific, Waltham, MA, USA) was used for ultra performance liquid chromatography (UPLC) and connected to the Q-Exactive hybrid quadrupole-Orbitrap mass spectrometer (Thermo Fisher Scientific) with a nano-electrospray ionization source. Labeled samples were injected into UPLC and concentrated in a C18 reverse phase trap column (100 µm I.D. × 5 mm length, Thermo Fisher Scientific) at a flow rate of 4 µL/min. Samples were then separated using a C18 reversed phase column (75 μm I.D. × 150 mm long, Nikkiso Tecnos, Tokyo) at a flow rate of 300 nL/min. A fixed mobile phase condition to Solvent A was water containing 0.1% formic acid and to Solvent B was acetonitrile containing 0.1% formic acid. Peptides were ionized by the positive mode of nano-electrospray ionization, which was a 1.8-kV capillary voltage.

Protein data were analyzed using Mascot Distiller v2.4 (Matrix Science, London, UK) http://www.matrixscience.com/mascot_support_v2_4.html, and a list of peaks was generated based on the recorded fragmentation spectra. In the identification of this protein, the UniProt amino acid sequence database (released on 4 March 2015: http://www.uniprot.org/) with biological species limited to the mouse (NCBI Taxonomy ID: 10090) and herpesviridae (ID: 10292) was used. The identified proteins were checked using the UniProt-GOA database (v140, http://www.ebi.ac.uk/GOA), and the annotation of Gene Ontology was added. Qualitative data and quantitative values were assessed using Mascot v2.4. In protein identification, the criteria of false rates of detection were less than 1%. The mass error was 10 ppm in Precursor Mass, and 0.01 Da in Fragment Mass. Due to the semi-quantitative nature of this approach, proteins were classified into three categories based on the values of iTRAQ quantitation (<5, low abundance; 5 to 10, medium abundance; and >10, high abundance) [46].

### 4.4. Statistical Analysis

Statistical analyses were performed using the Student’s *t*-test with Microsoft Excel (Microsoft, Redmond, WA, USA). Results were expressed as the mean ± SD. Differences were considered to be significant at *p* < 0.05.

## 5. Conclusions

Our results indicate that a HSV-1 RH2 infection increases the release of danger signal proteins and viral gene products, but decreases the release of ECM components. These changes may alter the tumor microenvironment and promote anti-tumor immunity against SCC.

## Figures and Tables

**Table 1:** Up-regulated proteins in the abundance group of secretomes ^a^. ^a^ Proteins up-regulated by more than 1.4 fold by the strain RH2 infection are listed. ^b^ Mean of three assessments. * *p* < 0.05.

Accession No.	Protein Name	MW (kDa)	iTRAQ Quantitation ^b^		Fold Change	*p*-Value
			Med24	MedC		
Q9QXS1	Plectin	534	79.7	54	1.48	0.057
B7FAU9	Filamin, alpha	280	73.7	49.7	1.48	0.004 *
P68372	Tubulin beta-4B	50	54.7	37	1.48	0.0027 *
Q9JKF1	Ras GTPase-activating-like protein IQGAP1	189	47.7	33.7	1.42	0.09
Q02053	Ubiquitin-like modifier-activating enzyme 1	118	40	28	1.43	0.058
Q5SXR6	Clathrin heavy chain	192	40	26.3	1.51	0.023 *
P80314	T-complex protein 1 subunit beta	57	39	23.7	1.65	0.012 *
P63038	60-kDa heat shock protein, mitochondrial	61	32.4	20.3	1.59	0.22
P42932	T- complex protein 1 subunit theta	60	31.3	20.3	1.54	0.0013 *
Q9CWJ9	Bifunctional purine biosynthesis	64	27.7	17	1.63	0.021 *
	protein PURH					
P11983	T-complex protein 1 subunit alpha	60	25.7	16.7	1.54	0.0088 *
P56480	ATP synthase subunit beta, mitochondrial	56	24	17	1.41	0.42
Q64737	Trifunctional purine biosynthetic protein	108	22.7	16	1.42	0.12
	adenosine-3					
Q9JHU4	Cytoplasmic dynein 1 heavy chain 1	532	26.7	12	2.22	0.16
D3YW87	Filamin-C	289	33.3	20.7	1.61	0.033 *
P80318	T-complex protein 1 subunit gamma	61	22.7	13.3	1.7	0.0078 *
P80315	T-complex protein 1 subunit delta	58	19.3	13.3	1.45	0.016 *
Q7TPV4	Myb-binding protein 1A	152	21.3	11.3	1.56	0.064
Q8CGC7	Bifunctional glutamate/proline-tRNA ligase	170	19.7	11.3	1.74	0.033 *
Q03265	ATP synthase subunit alpha, mitochondrial	60	19.3	10.3	1.87	0.17
P23116	Eukaryotic translation initiation factor 3	162	17	11.3	1.5	0.078
	subunit A					
Q78PY7	Staphylococcal nuclease domain-containing	102	16.7	11	1.51	0.12
	protein1					
B1AX58	Plastin-3	72	16	11.3	1.41	0.00015 *
Q9D8N0	Elongation factor 1-gamma	50	16.7	10.3	1.61	0.18
Q8BGQ7	Alanine--tRNA ligase, cytoplasmic	107	15.7	10.3	1.51	0.21
Q61553	Fascin	55	15	10.3	1.45	0.049 *
Q8CIE6	Coatomer subunit alpha	138	14.7	10	1.47	0.025 *
P21550	Beta-enolase	47	38	25.3	1.5	0.002 *
Q922F4	Tubulin beta-6 chain	50	27.7	17.7	1.57	0.011 *

**Table 2:** Down-regulate proteins in the abundance group of secretomes ^a^. ^a^ Proteins down-regulated by less than 0.5 fold by the RH2 infection are listed. ^b^ Mean of three assessments.

Accession No.	Protein Name	MW (kDa)	iTRAQ Quantitation ^b^		Fold Change	*p*-Value
			Med24	MedC		
A0A087WR50	Fibronectin	263	33.7	99.0	0.34	0.00032
P35441	Thrombospondin-1	130	15.7	48.3	0.32	0.00045
E9PZ16	Basement membrane-specific heparan	470	9.3	50.0	0.19	0.0011
	sulfate proteoglycan core protein					
P97298	Pigment epithelium-derived factor	46	16.7	42.7	0.39	0.0011
A2AQ53	Fibrillin-1	312	10.0	41.7	0.24	0.0000830
Q3UQ28	Peroxidasin homolog	165	6.7	36.3	0.18	0.0019
P33434	72-kDa type IV collagenase	74	11.0	31.3	0.35	0.0074
E9PZ00	Prosaposin	61	9.0	28.3	0.32	0.0016
D3Z598	Latent-transforming growth factor beta-binding protein 4	167	6.0	29.7	0.2	0.00022
Q07797	Galectin-3-binding protein	64	7.3	25.7	0.29	0.00022
P28653	Biglycan	42	4.7	27.7	0.17	0.0011
P10605	Cathepsin B	37	10.0	21.3	0.47	0.023
Q00493	Carboxypeptidase E	53	10.3	21.0	0.49	0.011
Q8VHY0	Chondroitin sulfate proteoglycan 4	252	6.3	25.0	0.25	0.0015
Q06890	Clusterin	52	6.3	23.3	0.27	0.00044
Q9R118	Serine protease HTRA1	51	7.0	22.3	0.31	0.00078
P07214	SPARC	34	6.3	22.7	0.28	0.00029
Q61398	Procollagen C	50	4.7	24.3	0.19	0.0000940
Q9QZF2	Glypican-1	61	7.3	19.7	0.37	0.0078
Q640N1	Adipocyte enhancer-binding protein 1	128	5.7	20.7	0.27	0.0004
Q8BND5	Sulfhydryl oxidase 1	83	5.3	21.0	0.25	0.0018
Q02819	Nucleobindin-1	53	7.7	17.3	0.44	0.016
Q9R0E2	Procollagen-lysine, 2-oxoglutarate	84	5.7	17.7	0.32	0.0037
	5-dioxygenase 1					
Q61468	Mesothelin	69	6.3	16.7	0.38	0.00039
M0QWP1	Agrin	217	2.7	18.3	0.15	0.00000480
Q61508	Extracellular matrix protein 1	63	3.0	17.7	0.17	0.0014
O08665	Semaphorin-3A	89	5.3	15.0	0.36	0.0054
Q9WVH9	Fibulin-5	50	2.3	18.0	0.13	0.00071
Q60963	Platelet-activating factor acetylhydrolase	49	2.3	17.7	0.13	0.00037
O88325	Alpha-N-acetylglucosaminidase	83	3.0	15.7	0.19	0.00046
P21460	Cystatin-C	16	5.3	13.3	0.34	0.0058
P55065	Phospholipid transfer protein	54	0.7	18.0	0.04	0.00048
P25785	Metalloproteinase inhibitor 2	24	5.7	12.7	0.45	0.012
Q8QZR4	Out at first protein homolog	32	4.7	13.7	0.34	0.0049
G3XA35	MCG116562, isoform CRA_a	263	4.7	13.3	0.35	0.007
Q8CE08	Prostatic acid phosphatase	44	2.7	15.3	0.17	0.0000700
P41245	Matrixmetalloproteinase -9	81	3.0	14.7	0.2	0.0013
P12023	Amyloid beta A4 protein	87	3.3	13.3	0.25	0.0042
P18242	Cathepsin D	45	5.0	11.7	0.43	0.034
Q9WV54	Acid ceramidase	45	2.7	14.0	0.19	0.00071
P08121	Collagen alpha-I (III) chain	139	2.0	13.3	0.15	0.011
G3UWC2	N-acetylated alpha-linked acidic dipeptidase 2, isoform CRA_a	87	1.7	11.7	0.14	0.0013
Q62165	Dystroglycan	97	2.3	10.3	0.23	0.00043
Q62356	Follistatin-related protein 1	35	2.3	10.0	0.23	0.0019
P11087	Collagen alpha-1(I) chain	138	2.0	10.3	0.19	0.011
Q9EPL2	Calsyntenin-1	109	0.7	11.3	0.06	0.0015
O88207	Collagen alpha-1(V) chain	184	0	12.0	0	0.00048
Q04857	Collagen alpha-1 (VI) chain	108	0	12.0	0	0.0000310
O09159	Lysosomal alpha-mannosidase	115	0	10.0	0	0.0000652
P47880	Insulin-like growth factor-biding protein 6	25	0	10.0	0	0.00098

**Table 3:** DAMPs and DAMPs-related proteins in secretomes. ^a^ Mean of three assessments. * *p* < 0.05.

Accession No.	Protein Name	MW (kDa)	iTRAQ Quantitation ^a^		Fold Change	*p*-Value
			Med24	MedC		
DAMPs						
P63017	Heat shock cognate 71-kDa protein	71	68.0	63.7	1.07	0.41
P11499	Heat shock protein HSP 90-beta	83	66.7	56.7	1.18	0.046 *
P60710	Actin, cytoplasmic 1	42	49.0	36.0	1.36	0.027 *
Q3U2G2	Heat shock 70 kDa protein 4	94	36.0	30.7	1.17	0.071
P07901	Heat shock protein HSP 90-alpha	85	57.7	49.7	1.16	0.052
P10107	Annexin A1	39	30.0	29.0	1.03	0.75
P10853	Histone H2B type 1-F/J/L	14	27.3	29.3	0.93	0.71
P63038	60-kDa heat shock protein, mitochondrial	61	32.3	20.3	1.59	0.22
P16045	Galectin-1	15	22.3	19 .0	1.18	0.067
P07356	Annexin A2	39	15.3	14.3	1.07	0.72
Q61171	Peroxiredoxin -2	22	15.0	13.7	1.10	0.33
O08709	Peroxiredoxin -6	25	11.7	9.7	1.21	0.18
Q9JMH6	Thioredoxin reductase 1, cytoplasmic	67	10.7	8.3	1.28	0.28
Q61699	Heat shock protein 105 kDa	96	12.3	8.0	1.54	0.25
P16110	Galectin-3	28	10.0	5.7	1.67	0.031 *
P99029	Peroxiredoxin-5, mitochondrial	22	9.0	6.3	1.42	0.091
P63158	High mobility group protein B1	25	5.7	4.7	1.22	0.66
P15864	Histone H1.2	21	3.0	7.0	0.43	0.0081 *
P10639	Thioredoxin	12	5.7	3.7	1.55	0.10
P14211	Calreticulin	48	3.7	3.3	1.12	0.90
DAMPs-related proteins						
Q3V117	ATP-citrate synthase	121	28.0	21.0	1.33	0.025 *
P56480	ATP synthase subunit beta, mitochondrial	56	24.0	17.0	1.41	0.42
Q03265	ATP synthase subunit alpha, mitochondrial	60	19.3	10.3	1.87	0.17
Q9DB20	ATP synthase subunit O, mitochondrial	23	4.7	4.0	1.17	0.80

**Table 4:** HSV-1 proteins in secretomes. ^a^ Mean of three assessments. UL, unique sequence of the L component; US, unique sequence of the S component; ICP, infected cell protein; VP, virion polypeptide.

Accession No.	Protein Name	MW (kDa)	Gene	iTRAQ Quantitation ^a^
				Med24
F8RG70	Ribonucleoside-diphosphate reductase large subunit, ICP6	124	UL39	33.7
D3YP93	Single-stranded DNA-binding protein, ICP8	128	UL29	32.7
L0N5H2	DNA polymerase processivity subunit	51	UL42	19.3
B9VQC5	Transcriptional regulator ICP4	133	α4	7.7
D3YP82	Major capsid protein, VP5	149	UL19	14.3
A0A0B5E4C5	UL12, Exonuclease	67	UL12	13.0
D3YPB9	Multifunctional expression regulator, ICP27	55	UL54	13.0
A0A0F7GRC1	Deoxyuridine triphosphatase	39	UL50	10.0
F8RCG6	Tegument protein UL37	121	UL37	10.0
A0A0B5E4D5	Thymidine kinase	41	UL23	8.3
A0A0B5E4F4	VP13/VP14	74	UL47	7.7
D0V7M2	Capsid protein VP23	34	UL18	7.7
A2I996	Glycoprotein D	43	US6	6.7
L0N6C7	Envelope glycoprotein E	59	US8	5.3
P10224	Ribonucleoside-diphosphate reductase small subunit	38	UL40	6.0
F8RCR4	Tegument protein VP11/12	78	UL46	5.3
F8RDC2	DNA packaging tegument protein UL25	63	UL25	5.0
L0N3F0	Tegument protein VP22	32	UL49	4.3
D3YPB2	Transactivating tegument protein VP16	54	UL48	3.7
A0A0F7CY40	Nuclear egress membrane protein	30	UL34	4.7
A0A0F7GTB2	Capsid maturation protease	67	UL26	1.7
A0A089YQM2	Glycoprotein B	100	UL27	1.7
A0A089YQK4	UL2, Uracil-DNA glycosylase	36	UL2	1.3
F8RFA5	Tegument protein UL21	58	UL21	1.3
A0A0B5EE83	Glycoprotein I	41	US7	1.0
D3YP85	Envelope glycoprotein H	90	UL22	0.7
L0N3E3	Envelope glycoprotein C	55	UL44	0.7
L0N6J6	DNA polymerase	137	UL30	0.7
A0A089WZ14	Type II membrane protein	18	UL45	0.7
